# Impact of Virtual Reality on Healthcare Provider Empathy for Older Adults With Sensory Impairment

**DOI:** 10.1093/geroni/igab046.235

**Published:** 2021-12-17

**Authors:** Suzanne Dutton

**Affiliations:** Sibley Memorial Hospital/Johns Hopkins, washington, District of Columbia, United States

## Abstract

Virtual reality (VR) is an innovative technology that can simulate dual sensory impairment so that healthcare providers and others can experience this affliction common in older adults. This study investigated whether VR simulation could increase empathy among healthcare workers. Healthcare providers experienced a 7-minute scenario from the viewpoint of “Alfred”, a 74-year-old with macular degeneration and high frequency hearing loss on a commercial VR headset (Oculus Rift). Using a one-group pre/post-test study design, we measured knowledge, changes in empathy, and assessed participants’ self-reported behavior change. Results showed that participants increased their knowledge and that 9 of 14 empathy items had statistically significant increases. Additionally, 97% of participants agreed or strongly agreed that they would utilize the information learned in their work with patients. In conclusion, evidence suggests VR is an effective intervention to increase empathy and positively change behavior to support persons with sensory impairment.

